# Autism Spectrum and Other Neurodevelopmental Disorders in Children of Immigrants: A Brief Review of Current Evidence and Implications for Clinical Practice

**DOI:** 10.3389/fpsyt.2021.566368

**Published:** 2021-03-18

**Authors:** Heiko Schmengler, David Cohen, Sylvie Tordjman, Maria Melchior

**Affiliations:** ^1^INSERM U1136, Institut Pierre Louis d'Epidémiologie et de Santé Publique, Sorbonne Université, Paris, France; ^2^École des Hautes Études en Santé Publique, Rennes, France; ^3^Department of Interdisciplinary Social Science, Utrecht Centre for Child and Adolescent Studies, Utrecht University, Utrecht, Netherlands; ^4^Department of Child and Adolescent Psychiatry, Reference Centre for Rare Psychiatric Diseases, Groupe Hospitalier Pitié-Salpêtrière, Assistance Publique Hôpitaux de Paris, Sorbonne Université, Paris, France; ^5^Institute for Intelligent Systems and Robotics, CNRS UMR 7222, Sorbonne Université, Paris, France; ^6^Pôle Hospitalo-Universitaire de Psychiatrie de l'Enfant et de l'Adolescent, Université de Rennes 1 and Centre Hospitalier Guillaume-Régnier, Rennes, France; ^7^Integrative Neuroscience and Cognition Center, CNRS UMR 8002 and University of Paris, Paris, France

**Keywords:** developmental disabilities, autism spectrum disorder, child development, migration, neurodevelopment, migrant health, maternal and child health

## Abstract

Children of immigrants may have higher neurodevelopmental risks than those of non-immigrant populations. Yet, some evidence suggests that this group may receive late diagnosis, and therefore miss beneficial early interventions. Clinicians may misattribute symptoms of disorders to other social, behavioral or language problems. Likewise, there might be cultural differences in parents' likelihood of perceiving or reporting first developmental concerns to clinicians. Population-based standardized screening may play an important role in addressing ethnic inequalities in the age at diagnosis, although further research focusing on cross-cultural use is necessary. Once children are diagnosed, clinicians may rely on culturally sensitive procedures (translation services, cultural mediators) to increase the accessibility of interventions and improve adherence among immigrant families. In this brief review, we provide an overview about what is currently known about the epidemiology and risk factors of neurodevelopmental disorders, paying special attention to autism spectrum disorder (ASD), in children of immigrants and suggest the necessity of population-based screening and culturally sensitive care.

## Introduction

There is increasing evidence based on literature reviews that children of immigrants are at higher risk of neurodevelopmental disorders, particularly autism spectrum disorder (ASD) (1–4). Other neurodevelopmental disorders, such as attention deficit hyperactivity disorder (ADHD), intellectual disability, and specific learning disabilities, have been studied less frequently and results are less consistent, with some studies reporting higher risks and others reporting lower or equal risks (5–8). Some evidence suggests that children of immigrants may face delays in diagnosis and increased difficulties in accessing appropriate health care (9–11). Especially in relation to ASD this is very concerning, as children of immigrants may be at risk of missing early interventions, which have been shown to be highly beneficial in terms of clinical prognosis (12). In this brief review, we provide a short overview on what is currently known about inequalities in neurodevelopmental health in children of immigrants and discuss implications for screening and clinical practice, paying special attention to ASD.

## Epidemiology of Neurodevelopmental Disorders in Children of Immigrants

Increased risks of ASD in children of immigrants were first reported in Sweden, the UK, and Australia as early as the 1970 and 1980s (3, 13), and have since been confirmed in various countries (1–4). The main results are summarized in Table 1. This table does not present an exhaustive list of all studies on ASD in children of immigrants.

**Table 1:** Studies on ASD in children of immigrants [based on Augereau et al. (14)].

**References**	**Population**	**Sample**	**Main results**
Gillberg et al. (13)	Epidemiological study based on a Swedish population registry comparing migrant parents' frequency between the autism and control group	35 children with autism (20 living in urban area and 15 living in rural area) compared to 42,886 age-matched children in the general population from the same region	30% of urban children with autism have migrant parents 0% of rural children with autism have migrant parents No significant difference regarding migrant parents' frequency between urban ASD and typically developing control groups Significantly lower frequency of migrant parents in rural ASD group, as compared to typically developing controls (p <0.001)
Gillberg et al. (15)	Study of autism prevalence in children born in Sweden from mothers born in Uganda	3 children with autism born in Sweden from mothers born in Uganda	Autism prevalence in children born in Sweden from mothers born in Uganda (15%) compared to the prevalence of autism in the general population (0.08%)
Gillberg et al. (16)	Epidemiological study based on a Swedish population registry comparing migrant parents' frequency between an autism and a control group	55 children with autism for 78,106 children in general population	27% of children with autism compared to 26.2% of children in general population have migrant parents (non-significant)
Lauritsen et al. (17)	Epidemiological study based on a Danish population registry comparing migrant parents' frequency between an autism and a control group	818 children with autism born in Denmark from a cohort of 943,664 children in general population	Relative risk of ASD evaluated at 1.42 when mothers were born outside European continent compared to mother born in Denmark
Keen et al. (18)	Retrospective study based on English clinical registry comparing ASD prevalence according to mother's native country and her ethnic group	428 children with autism	Relative risk of autism higher for children whose mothers were born in: - the Caribbean (RR in Lambeth = 10.01, 95% CI: 5.53–18.1; RR in Wandsworth = 8.89, 95% CI: 5.08–15.5); - Asia (RR in Lambeth = 3.97, 95% CI: 2.01–7.84; RR in Wandsworth = 2.08, 95% CI: 1.33–3.25); - Africa (RR in Lambeth = 7.92, 95% CI: 5.39–11.6; RR in Wandsworth = 3.27, 95% CI: 2.36–4.53).
Barnevik-Olsson et al. (19)	Epidemiological study based on a Swedish population registry focusing on autism prevalence in the Somali background population compared to the non-Somali population	−250 children with autism (232 non-Somali and 18 Somali) - 113,391 children in general population (111,555 non-Somali and 1,836 Somali)	Autism prevalence was higher in Somali background children compared to non-Somali background children (*p* < 0.001)
Magnusson et al. (20)	Case-control study based on register-based cohort (*Stockholm Youth Cohort*) comparing migrant parents' frequency between an autism and a control group	3,918 children with autism (2,269 high-functioning and 1,649 low-functioning) for 589,114 children in general population	Odds of low-functioning autism were higher in migrant population when both parents were born abroad (OR = 1.5, 95% CI: 1.3–1.7), mainly if parents came from countries with low Human Development Index (HDI)
Schieve et al. (21)	Study based on an American population registry comparing ASD prevalence in US-born non-Hispanic White children and US-born Hispanic children	US-born non-Hispanic White children (*n* = 37,265) and US-born Hispanic children (*n* = 4,690)	Significantly higher ASD prevalence (1.19%) in US-born non-Hispanic White children, compared to US-born Hispanic children with foreign-born parents (0.31%)
Becerra et al. (22)	Epidemiological study based on an American population registry comparing autism prevalence according to mother's native country and ethnicity	7,540 children with autism from a cohort of 1,626,354 children in general population matched on age	Higher relative risk of autism when mothers were born in the Philippines (RR = 1.23, 95% CI: 1.08–1.40) or in Vietnam (RR = 1.45, 95% CI: 1.24–1.70), compared to White mothers born in the USA
Abdullahi et al. (8)	Register-based study on the prevalence of ASD in children born to immigrant mothers in Australia	Western Australian population-based linked data of 764,749 singleton live births from 1980 to 2010	From 1980 to 1996, but not from 1997 to 2010, children born to mothers born in low-income countries had an increased relative risk of ASD with intellectual disability
Augereau et al. (14)	Study on parents' and grandparents' migrant status in French children with ASD and French children without ASD but with language disorders	30 prepubertal male children with ASD and 30 prepubertal male children without ASD but with language disorders (control group)	Absence of significant difference between the ASD and control groups for parental migration (migratory trip and post-migration experience), whereas HDI values of native countries were significantly lower for immigrant parents and grandparents in the ASD group, compared to the control group

Although there is variability in the strength of the reported associations, systematic reviews have found support of increased risks of ASD in children of immigrant parents (1–4). Particularly maternal immigrant status has been reported as an environmental risk factor for ASD (23, 24). Parental immigrant status seems to be mainly associated with low-functioning ASD (20) and other ASD phenotypes characterized by intellectual disability and communication disorders (25). Indeed, studies reported generally lower risks of high-functioning ASD (20) and Asperger's syndrome (26) in children of immigrants (25, 27), as compared to the host population. Neurodevelopmental disorders other than ASD have been examined less frequently, and results are less consistent. When considering ADHD, divergent results have been found, partially due to differences in informants, as well as in the accessibility of mental healthcare across countries. In Finland, Lehti et al. found higher odds of ADHD in children of immigrants in a case-control study (5). Similarly, in Germany, immigrant parents reported more ADHD symptoms in their children (28) and stronger increases in symptoms over time (29). However, German children born to immigrants had lower odds of receiving a diagnosis of ADHD (7). In Sweden, only non-European immigrants reported more symptoms of ADHD in their children (30). In Denmark, teachers reported similar numbers of ADHD symptoms in children of immigrants and non-immigrants, whereas immigrant parents reported lower levels of symptoms among their children (31). In Belgium, adolescents with a migrant background tended to self-report fewer symptoms of ADHD than their non-immigrant peers (32), whereas no differences in self-reported symptoms were found in Norway (33). In the Netherlands, Zwirs et al. found lower prevalence estimates of ADHD in children of immigrants, as compared to those of the native Dutch (34). In the USA, several population-based studies found lower risks of receiving an ADHD diagnosis in ethnic minority groups, including Hispanic Americans (35–41). However, a study specifically focusing on the Public Mental Health System of New York State found higher prevalences of ADHD in Hispanics and non-Hispanic Blacks than in Whites (42). Importantly, it must be noted that in the US, many studies on ethnic minority groups did not distinguish between immigrants and non-immigrants (e.g., in the case of Blacks or Hispanics), and few provide detailed information on individuals' country of origin (22).

Also when considering intellectual and learning disabilities, divergent results were observed. In Australia, Abdullahi et al. found higher prevalence rates of cerebral palsy with ID in children of mothers born in upper-middle-income countries only. They did not find evidence of increased risks of ID without cerebral palsy or cerebral palsy without ID in children of immigrant mothers (8). In two other Australian studies, mothers born abroad had no significant differences in their risk of having a child with severe ID, and when considering mild to moderate ID, foreign-born women tended to have lower risks (43, 44). A British study found lower prevalence rates of intellectual disabilities in children of immigrants, except those of Bangladeshi descent, who had higher odds of profound multiple learning difficulties, of Pakistani descent who had higher odds of profound multiple and severe learning difficulties, as well as Travelers of Irish heritage, who had significantly higher odds of moderate and severe learning difficulties (45). In France, the prevalence of severe ID was higher in areas with a high proportion of immigrants (46). In Finland, Lehti et al. found that children of immigrant parents had higher odds of developmental disorders related to speech and language, academic skills and coordination (6). Similarly, a German study found that children with foreign nationalities had higher odds of delays in gross motor skills, fine and grapho-motor coordination, grammar, memory and concentration, perseverance, abstraction, visual perception and arithmetic (47). In the US, two studies found no differences in the odds of learning disabilities in children of Latinos and Whites (37, 48), and a third study found lower odds of developmental delay, learning disabilities, and speech problems in children of immigrants (49). A Taiwanese study found lower risks of developmental delays in children of immigrants (50).

Inequalities in neurodevelopmental health may already be present at a very young age, long before children are typically diagnosed, though currently few studies on very young infants are available. In a population-based birth cohort in France, Schmengler et al. found that immigrant mothers more frequently observe early signs of neurodevelopmental problems in their 2-year-old children, as measured by a standardized parental-report screening instrument [Modified Checklist for Autism in Toddlers (M-CHAT)], compared to non-immigrant French mothers (51). In a recent Italian study on pre-term infants, children of migrant mothers from countries with a low Human Development Index (HDI) showed increased risks of general neurodevelopmental impairment at 24 months, as determined by the Revised Griffiths Mental Development Scale 0–2 years, particularly if they were exclusively fed with formula milk (52).

The timing and circumstances of migration seem to play an important role in neurodevelopmental inequalities in immigrant populations. For example, Magnusson et al. (20) reported that recent migration was particularly associated with risks of low-functioning ASD in the offspring, and children of mothers who migrated during the year they gave birth had the greatest risks. However, elevated risks are also observed in children of descendants of immigrants, which may suggest transgenerational effects of migration (22, 51). Importantly, risks differ strongly by parental region of origin. For example, in the USA, children of women born in the Philippines, Vietnam, and Central/South America have increased risks of ASD, compared to children of US-born White mothers, whereas no higher risks were found in children of women born in China, Japan, Korea, or Mexico (22). In France, children of mothers born in North and French-speaking Sub-Saharan Africa scored significantly higher on the M-CHAT than children of non-immigrant French mothers. However, no differences in M-CHAT scores were found when comparing children of non-immigrant French women and those born in other EU countries (51). In the UK, higher risks of intellectual and learning disabilities were mainly found in children of immigrants from Bangladesh and Pakistan, while most other immigrant groups had lower or similar risks of intellectual and learning disabilities, as compared to the non-immigrant British (45). Lehti et al. found higher odds of childhood autism in children of mothers and fathers born in Asia, the former Soviet Union, and former Yugoslavia. No higher odds were observed in children of parents born in Western countries, North Africa and the Middle East, and Sub-Saharan Africa (53). When considering ADHD, maternal migration was only associated with higher odds for children of mothers born in the former Soviet Union and Yugoslavia, Sub-Saharan Africa, and North Africa and the Middle East, whilst no higher odds were found in children of women born in Asia, Latin America, and Western countries. Paternal migration was associated with increased odds of ADHD regardless of fathers' region of birth (5). Regarding developmental disorders of speech and language, academic skills or coordination, Lehti et al. (6) found higher odds in children of women born in the former Soviet Union and former Yugoslavia, Sub-Saharan Africa, North Africa and the Middle East, Asia, and Latin America, but not other Western countries. Paternal migration was associated with higher odds of these developmental disorders regardless of fathers' region of birth, except for men born in Latin America, where no significant differences were found with non-immigrant Finnish men (6).

## Differential Exposure to Neurodevelopmental Risk Factors

It is not clear which risk factors underlie the association between parental immigrant status and offspring neurodevelopment. Several explanations have been suggested, which include an increased prevalence of known pre-, peri-, and post-natal risk factors for neurodevelopmental disorders (54–58). For example, immigrants frequently differ from the host population on risk factors, such as obesity (59, 60), and low socioeconomic status, as well as associated risk factors, such as inadequate housing and poor nutrition (46, 61–65). Furthermore, they may be more frequently exposed to environmental pollutants, such as dioxin, PCBs, traffic-related air pollution, or heavy metals (53, 65–69). In France, children born to immigrant women of African descent have significantly higher blood lead levels than children of non-immigrant French mothers (70). Etchevers et al. hypothesized that this may partially be related to cultural habits involving the use of contaminated goods, such as imported lead-releasing ceramic cookware, herbs, or cosmetics, such as surma or kohl (70). Immigrant mothers may face language and cultural barriers, as well as discrimination when accessing prenatal care (71), which could lead to deferred treatment of pregnancy complications, which are a known risk factor of neurodevelopmental problems (55, 56, 58, 72). Indeed, pregnancy complications seem more common in some immigrant populations (73–75), yet were unable to explain higher risks of ASD in children of immigrant mothers in Sweden (20) and more early signs of neurodevelopmental problems children of immigrant mothers in France (51). Another common, yet controversial, hypothesis is that elevations in neurodevelopmental risk might be due to increased risks of vitamin D deficiency in darker skinned populations (3). Gestational vitamin D deficiency was associated with ASD-related traits at age 6 in a recent analysis of a Dutch multiethnic cohort (76). In a Swedish registry-based study, vitamin D-deficiency in pregnancy predicted ASD with, but not without, intellectual disability (77). In another Swedish study, Somali women were more likely to be vitamin D deficient than Swedish women. However, no significant differences were observed when comparing Somali mothers with and without children with autism (78). In the Netherlands, no elevation in ASD risk was found in groups known to have high levels of vitamin D deficiency, such as the Moroccan Dutch (79). Associations between vitamin D deficiency in pregnancy and increased risks of ADHD have also been reported in studies (80, 81). Other explanations focus on psychosocial stress experienced during the premigration, migration, or post-migration periods, which may adversely impact neural development of the fetus, for example through epigenetic mechanisms (82, 83). Immigrants sometimes flee from armed conflict or severe economic insecurity in their countries of origin, and may have high levels of exposure to combat, poverty and sexual violence (84). Common post-migration psychosocial stressors include acculturative stress and discrimination (85, 86).

Some evidence suggests that risks are highest if parents originate from regions with low human development, which can be considered as a proxy of the extent of adversity in the country of origin. In Finland, children of both immigrant mothers and fathers from countries with low HDI had higher odds of ADHD, compared to children of immigrant parents from countries with very high, high, or medium HDI (5). However, no such associations were found when considering developmental disorders of speech and language, academic skills or coordination (6). In Sweden, only children of mothers from medium and low HDI countries had significantly higher risks of low-functioning ASD, whereas no differences were found between children of immigrant mothers from countries with high or very high HDI and those of non-immigrant Swedish women (20). In Australia, children with ASD born to immigrants from lower income countries were more likely to have comorbid intellectual disability, and showed more social impairments and communication difficulties (87). In France, the HDI values of native countries were significantly lower for parents and grandparents of ASD boys compared to controls (parents and grandparents of non-ASD boys with language disorders), especially for paternal grandparents, whereas no differences were found between the two groups when considering only (grand) parental immigrant status. Furthermore, HDI levels from the paternal line (father and especially paternal grandparents) were negatively correlated with autism severity, particularly for social interaction impairments (14). The authors hypothesized that social adversity-related stress experienced during the premigration period, especially by paternal grandparents, may be a factor of vulnerability for ASD (14). Furthermore, associations between low human development and the incidence of schizophrenia (which is nowadays considered a neurodevelopmental disorder), including childhood-onset schizophrenia, have been observed in immigrant populations (88–91). Unfortunately, currently very little is known about neurodevelopmental health in countries with low human development, such as those in Sub-Saharan Africa (92, 93). Importantly, bilingualism does not seem to be a determinant of developmental problems in children with and without clinical disorders, such as ASD (94–98), nor is it able to explain associations between parental immigrant status and neurodevelopmental disorders (51, 87).

## Differences in the Detection of Neurodevelopmental Disorders, Health Care Access, and Treatment Retention

Most available literature on the neurodevelopment of immigrants' children is based on disease registries and case-control studies of children diagnosed with a clinical disorder, such as ASD. These studies consider often only one neurodevelopmental disorder (e.g., ASD), despite that comorbidity is the rule rather than the exception among neurodevelopmental disorders (99–101). Furthermore, these studies miss very young children or those that remain undiagnosed (51). This is especially problematic considering that there is some evidence of ethnic diagnostic/referral bias in clinical practice, as clinicians may incorrectly attribute symptoms of neurodevelopmental disorders to other social, behavioral or language problems (102). For example, in an experimental study, Dutch pediatricians were more likely to consider an ASD diagnosis when evaluating clinical vignettes of children of non-immigrant parents, as compared to vignettes of children of Moroccan and Turkish-origin parents (102). Diagnostic bias may be less problematic when studying ethnic inequalities in ADHD, as ADHD symptoms are featured in many behavioral questionnaires, such as the Child Behavior Checklist (CBCL) and the Strengths and Difficulties Questionnaire (SDQ) (103, 104). These instruments are routinely included in population-based cohort studies, which also include undiagnosed children. Nevertheless, in studies based on clinical samples, underdiagnosis can lead to underestimation of the true extent of neurodevelopmental inequalities in the population. In clinical practice, immigrants' children with ASD may be at higher risk of not accessing highly beneficial early interventions (105).

The extent to which late or under-diagnosis is an issue for children of immigrants is not entirely clear and may vary across settings, by type of condition, and clinical severity. In several studies from the USA, children of ethnic minorities, including African Americans and Hispanics were on average diagnosed later with ASD than children of White Americans or remained undiagnosed (106, 107), suggesting that developmental problems in children from these populations may systematically be missed. Other studies found no differences or yielded opposite associations. A Dutch population-based cohort study did not find differences in the age of ASD diagnosis between children of immigrants and non-immigrants (79), whereas Abdullahi et al. found that Australian children of immigrants were diagnosed with ASD at earlier ages than their non-immigrant peers (87). However, children of immigrants in Australia were more likely to show more severe ASD accompanied with intellectual disability (87). It is therefore possible that an earlier age at diagnosis in this group is mainly explained by a higher prevalence of comorbidities and more severe phenotypes that are easier to detect in clinical practice (87). Importantly, diagnostic bias may still be present concerning children with less severe or more ambiguous symptoms, which may, for instance, explain the underrepresentation of children of immigrants in studies of children diagnosed with high-functioning ASD (20).

Disparities may exist not only in the timing of diagnosis and initial contact to health care providers, but also concern retention in high-quality neurodevelopmental care. For example, in a US study, autistic children of Latino parents with limited English proficiency received fewer hours of therapy and had higher unmet therapy needs than non-Latino White children (108). In another US study, children with ASD from immigrant families had more than twice the risk of not having a usual source of care (9). However, a study from the UK did not find ethnic differences in ASD-related care use (109). Currently, less is known about inequalities in access to ASD-related care in other European countries, where data on ethnicity are often not routinely collected (110). Nevertheless, in France, two sociological studies conducted in districts of Paris (111) and Marseille (112) that are characterized by a high prevalence of immigrant families show increased difficulties in accessing adequate care. These difficulties occurred despite the fact that France has universal healthcare access and that the principle of justice/equity within the healthcare system is inscribed in law (113). This suggests that current efforts may still be insufficient to address disparities in access to neurodevelopmental care for immigrant families in France. When considering ADHD care, studies have consistently shown lower use of ADHD medication in children of immigrants (114). For example, a study on Dutch ADHD patients aged <19 years found that higher proportions of patients with Turkish or Moroccan backgrounds reported never having used ADHD medication compared to non-immigrant Dutch patients. Furthermore, these groups showed higher medication discontinuation rates (115). Higher rates of medication discontinuation and treatment disengagement have also been found in ethnic minorities in the US, including Latin Americans (116, 117).

## Barriers to the Detection of Neurodevelopmental Disorders, Health Care Access, and Treatment Retention

Potential barriers to diagnosis, access to and retention in specialized care reported in studies conducted among immigrants are similar across disorders and include both structural/socioeconomic factors (e.g., financial barriers, underinsurance, problems with transportation, fragmented services, and language barriers) and social/cultural factors (e.g., lack of social support, stigma and discrimination, insufficient understanding of the host country's health system, low health literacy, and differences in values and expectations between health service providers and parents) (9, 10, 116, 118–126).

This mismatch in expectations may often stem from the fact that cultures differ substantially in their perceptions of the etiology and symptoms of neurodevelopmental disorders, on their prognosis, as well as on the effectiveness and acceptability of treatments. For example, studies suggest that the likelihood that parents attribute certain behaviors to a medical condition differs across ethnic groups (125). Some behaviors typical of ASD, such as avoiding eye contact, may be perceived as a way of expressing respect toward authority figures rather than a sign of atypical development in some Asian cultures (11, 118, 125). Likewise, the extent to which children engage in imaginative play differs across cultural groups (125). For example, Korean-Americans may be less likely to engage their children in pretend play, as compared to White Americans (127). Hence, the absence of imaginative play may be a more salient warning sign in some cultures than others (125). Such cultural differences in norms of optimal child development and parenting may influence whether and when early warning signs are recognized and reported (125). In a US study, Latina mothers were less likely to report developmental concerns and ASD symptoms than White mothers of children potentially with ASD, although children of Latino mothers who met the diagnostic criteria for ASD tended to have more severe ASD symptoms (128). Somali parents in Britain correctly identified clinical vignettes of children with autism, but had difficulties recognizing other developmental problems, which they misclassified sometimes as ASD-like symptoms (129). Indian parents may be first concerned by social difficulties, whereas US parents notice first delays in language and general development (11). Ethnic differences in reporting were also found when considering ADHD, suggesting that immigrant parents may be less likely to identify symptoms of ADHD in their children. In the Netherlands, Moroccan, Turkish, and Surinamese parents showed lower sensitivity in correctly identifying ADHD in their children than non-immigrant Dutch parents. At the same time, Moroccan and Turkish parents showed higher specificity in detecting ADHD than the non-immigrant Dutch (130). In line with this, in a Danish study, immigrant parents reported fewer symptoms of ADHD than non-immigrant parents in their children, whilst no such differences were found when considering teacher report (31). This could be because cultures may differ in the extent to which they view behavioral problems typical of ADHD as a medical issue rather than a social or spiritual problem (42, 131). A US study found that traditional gender roles and cultural values of familism were related to sociological and spiritual beliefs about the etiology of ADHD amongst Latinos (131).

Furthermore, immigrant parents may have less support from friends and family networks, and may even face stigma from their own community, which can lead to social isolation of these parents (121). For example, in some cultures, having a child with ASD is perceived as a divine punishment or a consequence of evil spirits or witchcraft (118). Perhaps more frequently, communities are not aware of the challenges of raising a child with a long-term neurodevelopmental disability, such as ASD. In a study within the Somali community in Britain, many families were unfamiliar with the term “autism” when their child was diagnosed, as ASD is not recognized in Somalia and no corresponding word exists in Somali language (132). As a result of lack of ASD awareness, parents sometimes received conflicting messages from their community and medical professionals. For example, parents were often told by members of their extended family to disregard the diagnosis of ASD, as their child may grow out of the symptoms on his or her own (132). This led frequently to a confusion between ASD as a long-term diagnosis and temporary delays in development (132). Furthermore, some parents tended to delay accessing care and to hide their children from their community for fear of negative responses to the child's disruptive behavior (132). Also immigrant parents of children with ADHD may face difficulties finding social support from within their communities. For example, a qualitative study on US Latinos reported that families often blamed parents for problematic child behavior rather than viewing ADHD as a mental health condition. As a result, some parents had a tendency to conceal their child's condition to their extended family (133). Preferences concerning treatment modalities differ also across cultural groups. For example, Latino and African American parents tend to rate behavioral treatments for ADHD more favorably than White Americans, whilst being more reluctant to accept the use of psychotropic drugs on their children (134).

## Screening May Address Inequities in Access to Early Interventions for ASD

The use of standardized screening instruments in unselected populations may help address potential referral bias and improve early detection of neurodevelopmental disorders in children of immigrants and other underserved communities, although further research focusing on multiethnic contexts is necessary. Early detection is especially important in the case of ASD, as intervention in early childhood is a critical determinant of long-term clinical prognosis (12). Some initial results on the multiethnic use of standardized screeners in toddlers (e.g., M-CHAT) seem promising, though studies also have found differences in the performance of questionnaires across language and cultural groups. In a US study, children of ethnic minorities showed inflated screen-positive rates on the M-CHAT-R questionnaire. However, this effect disappeared when additionally considering the follow-up interview M-CHAT-R/F or subsequent clinical evaluations (135). No significant differences in the positive predictive values (PPVs) were found across ethnic groups for the M-CHAT-R with follow up (135), which was similar to the findings reported in another US study by Herlihy et al. (136). Furthermore, for children who screened at-risk for developmental delay based on the M-CHAT, Herlihy et al. found only small differences in age at evaluation between White and ethnic minority children, which suggests that standardized screening instruments may address inequalities in age at diagnosis (137). Seif-Eldin et al. found that the test performance of the M-CHAT in nine Arab-speaking countries was similar to Western contexts (138). However, some results from other studies were less encouraging, which highlights the importance of additional research on the cross-cultural validity and adaptation of neurodevelopmental screening instruments. For example, in the USA, Scarpa et al. reported poor internal consistency of the M-CHAT in children of mothers with ethnic minority background (139), and Guthrie et al. observed higher PPVs and specificity in children of Whites, as compared to those from other ethnic groups (140). Many other commonly-used ASD screeners have been assessed in terms of their cross-cultural validity, such as the Autism Spectrum Quotient (AQ)-Child questionnaire, the Social Responsiveness Scale (SRS), and the Social Communication Questionnaire (SCQ). Overall, studies support the cross-cultural use of these scales, though similar to the M-CHAT, some differences were found across settings. Carruthers et al. compared the predictive ability of individual items of the Autism Spectrum Quotient (AQ)-Child questionnaire across samples in Japan, India, and the UK. Whereas they detected cultural differences on some of the items, they found considerable cross-cultural overlap in the items that predicted most strongly ASD diagnosis (141). Wang et al. found similar sensitivity, specificity, and total scores in the Mandarin-adaptation of the social responsiveness scale (SRS) in Taiwan, compared to Western contexts (142). Similarly, Bölte et al. found satisfactory-to-good reliability and validity regarding the German adaptation of the SRS (143). However, whereas the raw SRS scores in controls in Taiwan and Germany were similar, these scores were lower than in controls from the US (142). Adequate performance of the SRS has also been found in South Korea (144) and for the Farsi adaptation of the scale in Iran (145). Studies that support the cross-cultural use of the Social Communication Questionnaire (SCQ) have been conducted in Taiwan (146), Greece (147), Germany (148), the Gulf countries (149), and Turkey (150). Detailed reviews on cultural adaptations of autism screening instruments have been performed by Soto et al. (151) and Al Maskari et al. (152).

Whereas current adaptations of screening instruments are often limited to language translations, the accuracy of these instruments could potentially be improved by making further adjustments to incorporate the norms, values, and beliefs of the adapted culture (152). For example, in Iran, Samadi and McConkey found that a screening instrument developed specifically for the Iranian context outperformed language adaptations of the M-CHAT (153). Additional difficulties relate to poor implementation of screening in clinical practice. For example, Rea et al. reported low screening completion rates and referral practices that were inconsistent with screening results in a retrospective medical chart review across three American pediatric clinics (154).

## Conclusion

Autism spectrum disorder (ASD) has by far been the most studied neurodevelopmental disorder in terms of its relation with parental migration. It is also the disorder for which inequalities in risk have been most consistently found (1–4). Less is known about other neurodevelopmental disorders, on which studies have found heterogeneous results, with some studies reporting higher and others lower risks in children of immigrants (5–8). Importantly, there is a profound lack of research on children of immigrants living in non-Western host countries. Differences in the exposure to environmental risk factors may contribute to inequalities in neurodevelopmental health between children of immigrants and non-immigrants. Although the contribution of these risk factors to the etiology of neurodevelopmental disorders is thought to be substantial (up to 40–50% in case of ASD), research in this area is still in its infancy (155). Common limitations of existing studies include, for example, lack of prospective longitudinal designs, small sample sizes, lack of direct measurement of environmental exposures, and difficulties ascertaining the timing of exposures (155). Particularly little is known about environmental risk factors in developing countries, which may differ substantially in their prevalence and distribution from high-income countries (92). Nevertheless, some environmental risk factors (e.g., ambient air pollution, certain pregnancy complications) have been repeatedly associated with neurodevelopmental disorders in studies (55, 155). Yet, the contributions of individual risk factors tend to be small and findings are not specific enough to derive interventions to prevent neurodevelopmental disorders that go beyond the provision timely diagnosis and adequate medical care (55). There is also a scarcity of studies on the experiences of immigrant families with children with neurodevelopmental disorders in the healthcare system, and, to our knowledge, there is currently no systematic review available summarizing what is currently known about these families' interactions with providers or the utility of existing interventions. Nevertheless, the limited available evidence suggests that many barriers to detection, health care access, and treatment retention are similar across neurodevelopmental disorders and include structural/socioeconomic factors, such as underinsurance and service fragmentation, as well as social/cultural factors, such as lack of social support from the same-ethnic community and insufficient availability of culturally appropriate care (9, 10, 116, 118–126).

Several recommendations can be made to improve diagnosis and access to neurodevelopmental healthcare for children of immigrants. When addressing disparities in the age of diagnosis, it is important to be aware that different cultural groups may not be equally likely to raise concerns about their child's development. Likewise, clinicians may misattribute certain symptoms to language difficulties or cultural differences (102). The implementation of standardized screening instruments in routine medical practice may be helpful as they prompt clinicians to look specifically for early signs of ASD and other neurodevelopmental disorders that otherwise may be missed (102). Screening at day-care centers, where many young children spend a significant amount of time, may also help address barriers in access to early detection and intervention (156). Childcare professionals could be trained to identify signs of neurodevelopmental problems and ASD as early as possible and inform parents in a culturally sensitive manner. Once children are diagnosed, clinicians should be encouraged to use culturally sensitive procedures (translation services, cultural mediators) to make treatment more accessible and enhance adherence among immigrant families (11, 157, 158). In settings where sufficient resources are not available, home-based early interventions may be helpful, which are implemented by caregivers in close collaboration with healthcare professionals (159).

## Author Contributions

HS and MM conceived and developed an initial draft manuscript in consultation with DC and ST, who regularly provided extensive feedback. All authors contributed to the final version of the manuscript.

## Conflict of Interest

The authors declare that the research was conducted in the absence of any commercial or financial relationships that could be construed as a potential conflict of interest.
